# Pattern Changes and Recurrent Remissions in Cervical Dystonia: Insights from a Long-Term Treated Case

**DOI:** 10.3390/toxins18060243

**Published:** 2026-05-25

**Authors:** Simone Aloisio, Massimiliano Passaretti, Luca Angelini, Martina De Riggi, Francesca Santachiara, Anna Sofia Grandolfo, Daniele Birreci, Matteo Bologna

**Affiliations:** 1 Department of Human Neurosciences, Sapienza University of Rome, 00185 Rome, Italy; simone.aloisio@uniroma1.it (S.A.); martina.deriggi@uniroma1.it (M.D.R.); francesca.santachiara@uniroma1.it (F.S.); annasofia.grandolfo@uniroma1.it (A.S.G.); daniele.birreci@uniroma1.it (D.B.); 2 Department of Translational and Precision Medicine, Sapienza University of Rome, 00185 Rome, Italy; 3 K8 Neuro Pereira, K8 Klinisk Neurovetenskap, Department of Clinical Neuroscience, Karolinska Institutet, 171 77 Stockholm, Sweden; massimiliano.passaretti@uniroma1.it; 4 IRCCS Neuromed, 86077 Pozzilli, Italy; luca.angelini@uniroma1.it

**Keywords:** cervical dystonia, botulinum toxin, pattern changes, remissions, torticollis, long-term follow-up

## Abstract

Cervical dystonia (CD) is increasingly recognized as a disorder with a dynamic clinical course rather than a fixed phenomenological profile. This report describes the long-term observation of a man with isolated CD followed for nearly 12 years from symptom onset. The course was characterized by multiple pattern changes, including a clear shift from left- to right-sided torticollis in 2019, followed by alternating and mixed dystonic patterns. Three remission phases were documented during follow-up: two prolonged periods in 2017–2018 and 2020–2021, and a further phase from March to November 2022. Over time, treatment requirements decreased from higher initial doses (500–700 U abobotulinumtoxinA and up to 130 U onabotulinumtoxinA) to lower maintenance doses of 30–50 U incobotulinumtoxinA/onabotulinumtoxinA, with longer injection intervals and no obvious documented need for dose adjustment to maintain clinical responsiveness. This case highlights the coexistence of pattern changes and recurrent remissions within a single continuously followed patient over an unusually long period. Although a treatment-related mechanism remains speculative and alternative explanations cannot be excluded, the observation underscores the value of prolonged follow-up and individualized treatment strategies in CD.

## 1. Introduction

Cervical dystonia (CD) is the most common form of focal dystonia and is characterized by sustained or intermittent involuntary muscle contractions leading to abnormal postures and movements of the head and neck [1]. According to current consensus classification, dystonia is defined as a disorder of patterned, repetitive, and often tremulous movements or postures, with CD typically classified as an isolated focal dystonia with variable temporal features, including fluctuations and possible remissions [2].

From a pathophysiological standpoint, CD is increasingly considered a disorder of distributed motor networks involving basal ganglia, cerebellum, and cortical regions, characterized by aberrant plasticity [3]. Consistent with this framework, neurophysiological studies have demonstrated aberrant sensorimotor plasticity in primary dystonia, and botulinum toxin (BoNT) has been shown to transiently reduce this abnormal response, suggesting that it may modulate central sensorimotor processing in addition to its peripheral chemodenervating effect [4]. Early electrophysiological studies also showed that dystonic muscle activity is not fixed but fluctuates over time, with variable co-contraction patterns even during stable postures, supporting the concept of an unstable and dynamically regulated motor program [5]. However, this should be considered only one possible explanatory model, as longitudinal clinical changes in CD may also reflect spontaneous remission, natural fluctuation, shifting dominance of muscle recruitment, or observer-dependent pattern classification during follow-up [6,7].

In parallel, longitudinal studies have shown that CD may exhibit pattern changes over time, with approximately one-third of patients developing pattern modifications during follow-up without clear evidence of reduced clinical responsiveness [8,9,10]. These findings challenge the traditional view of CD as a disorder with a stable clinical presentation. In this context, pattern change reflecting disease evolution should be distinguished from apparent change related to altered recognition of the dominant pattern during treatment, including possible unmasking of less evident motor configurations [8,9,10].

Remissions have also been described in a subset of patients with CD, although their underlying mechanisms remain incompletely understood. A systematic review and meta-analysis reported remission rates of approximately 15%, often followed by relapse, indicating that remission is part of the disease spectrum [11]. Emerging evidence suggests that remissions may, at least in some cases, be associated with BoNT therapy, raising the possibility that peripheral interventions may indirectly influence central motor networks and contribute to long-term changes in disease expression [12].

BoNT represents the gold standard treatment for CD, with well-established long-term efficacy and safety, although treatment requires continuous adjustment over time [13,14]. Increasing evidence suggests that long-term management is inherently dynamic, requiring ongoing individualization of injection patterns, doses, and intervals in response to dystonic pattern changes [15].

Despite these advances, longitudinal observations capturing the dynamic course of CD remain limited. Although pattern changes and remissions have been described separately, their integration within a single continuously followed patient has been only infrequently documented in the literature. Likewise, recurrent remissions within the same individual appear to have been only infrequently documented with comparable longitudinal and clinical detail.

This report describes a long-term case of isolated CD characterized by multiple pattern changes, recurrent remissions, and progressive reduction in BoNT requirements over nearly 12 years of follow-up.

## 2. Results

The patient, a 30-year-old man, presented with CD characterized by retrocollis and leftward deviation of the head. BoNT treatment was initiated in 2015, with an initial left-sided torticollis and partial clinical response. Following treatment optimization, the patient experienced progressive clinical improvement leading to a first prolonged remission between 2017 and 2018, during which neurological examination was normal and injections were withheld. In 2019, dystonic symptoms re-emerged, together with a clear pattern change from a left-sided to a right-sided torticollis. This new pattern persisted and later evolved into a more complex presentation including retrocollis and rightward rotation. Between 2020 and 2021, a second prolonged remission was observed, again characterized by minimal or absent dystonic features and temporary discontinuation of treatment. From 2021 onwards, the clinical picture became more variable, with re-emergence of left-sided features and subsequent fluctuations between left-, right-, and mixed-pattern dystonia. These changes occurred without an obvious documented loss of clinical responsiveness. During later follow-up, a further remission was observed from March 2022 to November 2022. Thereafter, symptoms remained generally mild on routine clinical examination, while BoNT doses were progressively reduced and injection intervals became longer.

Overall, the longitudinal course was marked by multiple pattern changes, including a clear lateralized shift from left to right and subsequent alternating and mixed dystonic configurations, together with three remissions—two prolonged (2017–2018 and 2020–2021) and a further interval from March to November 2022—and subjectively preserved clinical responsiveness despite progressive dose reduction.

**Figure 1:**
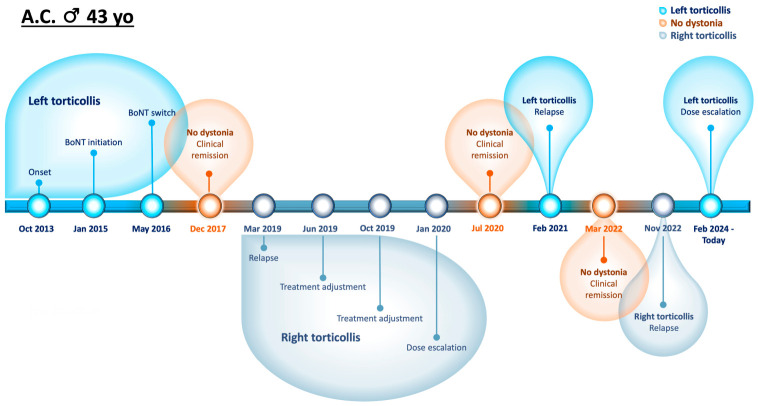
Clinical timeline of CD evolution over nearly 12 years, showing pattern changes, recurrent remissions, and progressive modulation of BoNT treatment. Left-sided dystonic patterns are highlighted in blue, right-sided dystonic patterns in grey-blue, and remission phases in orange. Each circle represents a main clinical timepoint, and labels identify onset, treatment initiation or adjustment, relapse, dose escalation, and remission.

A schematic representation of the clinical course is shown in Figure 1. The main selected clinical timepoints, corresponding dystonic patterns, and key BoNT treatment characteristics are summarized in Table 1, while the detailed longitudinal treatment chronology is provided in Supplementary Table S1. Representative examples of left-sided and right-sided dystonic phenotypes, together with a remission phase observed during follow-up, are shown in Video S1.

## 3. Discussion

The present case provides an informative longitudinal perspective on CD, highlighting the coexistence of multiple pattern changes, recurrent remissions, and progressive reduction in BoNT requirements, altogether compatible with a more dynamic disease model than traditionally assumed. A major strength of this report lies in the duration of clinical observation. While pattern changes, remissions, and non-linear disease trajectories have been described separately, detailed single-case longitudinal data spanning nearly 12 years from symptom onset remain limited [11,15,16]. Moreover, recurrent remissions within the same continuously followed individual appear to have been only infrequently documented with comparable longitudinal depth.

The occurrence of multiple pattern changes is a relevant feature of this report and further supports the dynamic nature of the disorder. Overall, the present case is broadly aligned with previous longitudinal observations showing that pattern change in CD may occur without compromising responsiveness to BoNT and tends to involve predominantly rotatocollis/torticollis and laterocollis phenotypes, particularly in relatively milder forms and within the first years of treatment [9,10]. Earlier studies also suggested that these modifications may reflect either recruitment of previously less relevant muscles during treatment or a more intrinsic reorganization of the abnormal central motor program [8]. More recent evidence has highlighted a possible contribution of altered afferent input, treatment-related cortical plasticity, peripheral and central BoNT effects, and an “unmasking phenomenon”, whereby treatment of the dominant dystonic pattern may reduce the overt expression of one motor configuration and reveal a previously less evident competing pattern [10]. In this respect, the present case appears broadly consistent with the available literature, as repeated pattern changes occurred over a prolonged course of follow-up and therapeutic modulation [8,9,10]. The salient characteristics of these previous reports, together with the main features of the present case, are summarized in Table 2.

Recurrent remissions represent another key element of this case. Although remission in CD is recognized as part of the disease spectrum and is often followed by relapse, it is generally described as an isolated event within cohort-based or retrospective series rather than as a recurring feature within the same continuously documented patient [11,12]. In the present report, remissions recurred over time in the context of longitudinal BoNT treatment, suggesting a dynamic and potentially reversible imbalance between pathological and compensatory mechanisms. However, this interpretation should remain cautious. While the occurrence of these remissions during the treatment course, together with progressive dose reduction and longer injection intervals, may be compatible with treatment-related modulation of sensorimotor networks, alternative explanations, including spontaneous fluctuation, cannot be excluded [11,12].

Taken together, the repeated pattern changes and recurrent remissions may represent different expressions of a shared process, consistent with current models of CD as a disorder of maladaptive plasticity within distributed motor networks. In this framework, the clinical variability observed in this patient may reflect fluctuating dominance among competing motor patterns over time [3,5]. At the same time, the present case cannot discriminate between true longitudinal reconfiguration of dystonic output and alternative explanations such as spontaneous fluctuation, regression toward a milder clinical expression, altered recognition of the dominant pattern during treatment, or observer-dependent classification over prolonged follow-up [6,7].

The contribution of BoNT warrants particular attention. Beyond its peripheral action at the neuromuscular junction, BoNT may influence central sensorimotor processing through modulation of afferent input. In the present case, the progressive dose reduction and longer duration of effect may suggest that long-term treatment may interact with adaptive plasticity mechanisms and contribute to reshaping disease expression over time, supporting a role beyond purely symptomatic peripheral control [13,14]. This interpretation is further supported by neurophysiological evidence showing that BoNT can transiently reduce the abnormally enhanced associative plasticity observed in primary dystonia, including in body regions distant from the injected muscles [4]. In addition, successful treatment may induce meaningful postural and biomechanical changes, further suggesting that BoNT can modify the clinical expression of CD beyond simple peripheral chemodenervation [17]. Altogether, these findings suggest the view that BoNT may influence central sensorimotor processing beyond peripheral chemodenervation, and that the dynamic course observed in our patient may reflect the interplay between intrinsic network instability and long-term therapeutic modulation of sensorimotor processing.

## 4. Conclusions

In conclusion, this case is consistent with the view of CD as a dynamic and treatment-responsive network disorder rather than a fixed phenomenological entity. Its main novelty lies in documenting, over a nearly 12-year follow-up, the coexistence of multiple pattern changes including alternating lateralization, recurrent remissions, and progressive reduction in BoNT requirements. While these longitudinal features may be compatible with effects of BoNT beyond symptomatic control, this interpretation remains speculative, and further investigations are needed to better define the mechanisms underlying these longitudinal changes. Overall, the case underscores the value of prolonged follow-up and individualized therapeutic strategies in CD.

## 5. Materials and Methods

This study consists of a retrospective longitudinal clinical observation of a single patient diagnosed with isolated CD and followed for more than ten years at the Movement Disorders outpatient clinic of Sapienza University of Rome. Serial neurological evaluations were performed during follow-up, including detailed characterization of dystonic phenomenology, with particular attention to predominant pattern, laterality, severity, and associated features such as pain and tremor. Follow-up outpatient visits were generally scheduled every 3–4 months. Clinical status and treatment response were assessed on the basis of routine neurological examinations performed during follow-up. No formal standardized longitudinal rating scales (e.g., TWSTRS, Tsui, CGI, structured pain rating, or blinded video scoring) were systematically collected, and no blinded adjudication was performed. Some video recordings were obtained during follow-up, after acquisition of written informed consent, to document and review the longitudinal clinical evolution. Disease classification followed current consensus criteria, including assessment of temporal features such as onset, variability, and course [2]. The patient received regular intramuscular injections of BoNT according to standard clinical practice [14]. Injections were performed under ultrasound and electromyographic guidance. BoNT dilution was 2 mL for onabotulinumtoxinA and incobotulinumtoxinA, and 2.5 mL for abobotulinumtoxinA. Treatment parameters included injected muscles, toxin formulation, total dose, and injection intervals. Adjustments were made over time based on clinical evolution, following established recommendations for CD management [14]. The main clinical timepoints, together with the corresponding total doses and selected target muscles, are summarized in Table 1. Pattern changes were defined as clinically evident modifications in the predominant dystonic pattern and/or laterality across follow-up visits. Remission phases were defined as periods of near-complete or complete resolution of overt dystonic symptoms on neurological examination, documented during follow-up after previous BoNT treatment and occurring in the absence of ongoing injections [11]. Given the retrospective descriptive nature of the case, no formal quantitative threshold or blinded adjudication was available. Relapse was defined as the re-emergence of clinically evident dystonic symptoms after a documented remission phase. Clinical data were analyzed descriptively.

## Figures and Tables

**Table 1:** Longitudinal summary of the main clinical timepoints, dystonic pattern, BoNT formulation, dose, and target muscles in the present case.

Timepoint	Age (Years)	Main Dystonic Pattern	BoNT Formulation	Total Dose	Target Muscles	Clinical Phase
October 2013	30	Retrocollis with leftward deviation	None	—	—	Onset
January 2015	32	Left torticollis with mild retrocollis	AboBoNT-A	500 U	Left SPL, right SCM	BoNT initiation
May 2016	33	Persistent left-sided pattern	IncoBoNT-A	100 U	Left SPL 50 U, right SCM 50 U	BoNT switch
December 2017	35	No overt dystonia	None	—	—	Clinical remission
March 2019	36	Mild right head tilt	IncoBoNT-A	75 U	Left SCM, left SPL	Right-sided relapse
June 2019	36	Right torticollis	IncoBoNT-A	100 U	Left SCM, left SPL	Dose escalation
October 2019	37	Retrocollis with rightward rotation	OnaBoNT-A	100 U	Right SPL 70 U, left SPL 30 U	BoNT switch
January 2020	37	Persistent right torticollis	OnaBoNT-A	130 U	Right SPL 70 U, left SCM 30 U, left SPL 30 U	Dose escalation
July 2020	37	Minimal rightward rotation	None	—	—	Clinical remission
February 2021	38	Mild left-sided recurrence	IncoBoNT-A	70 U	Left SPL 40 U, right SPL 30 U	Left-sided relapse
March 2022	39	No overt dystonia/ minimal residual signs	None	—	—	Clinical remission
November 2022	40	Mild rightward rotation during work activity	IncoBoNT-A	70 U	Right SPL 40 U, left SCM 30 U	Right-sided relapse
February 2024–today	41–43	Mild left tilt with rightward rotation	OnaBoNT-A	50 U	Left SPL 30 U, left SCM 20 U	BoNT switch; Stable treatment

Note: In cases where muscle-specific BoNT posology is not indicated, only the total injected dose was available in the clinical documentation.

**Table 2:** Comparison of the main studies addressing pattern changes in CD during BoNT treatment and the present case. For each study, the table summarizes study design and sample, main clinical-demographic features, frequency and timing of pattern change, most frequent patterns or conversions, and BoNT treatment characteristics.

	Kaňovský et al., 1997 [8]	Maia et al., 2010 [9]	Trinchillo et al., 2025 [10]	Present Case
Study	Secondary worsening after successful BoNT treatment in CD	Clinical changes of CD pattern in long-term BoNT-treated patients	Predisposing factors to pattern change in CD	Pattern changes and remissions in CD: insights from a long-term treated case
Design/sample	Electrophysiological/polymyographic study; 76 idiopathic CD patients treated with BoNT	Retrospective long-term cohort; 67 CD patients on regular BoNT	Single-center longitudinal study; 100 idiopathic CD patients treated with BoNT ≥ 3 years	Retrospective longitudinal single-case observation
Clinical-demographic features	Whole cohort: mean age 47.5 years; mean age at onset 43.4 years; mean disease duration 2.13 years. Non-responder subgroup: mean age 47.8 years; onset 41 years; disease duration 6.8 years.	Pattern change subgroup (*n* = 24): mean age at onset 33 years; mean dystonia duration 14.35 years; dystonia elsewhere in 37.5%; mean follow-up 80 months.	100 patients, 60 women; mean age 64.2 years; mean age at onset 47.9 years; mean disease duration 16.2 years; mean BoNT treatment duration 10.7 years; 80 focal and 20 segmental cases.	Male patient; age at onset 30 years; isolated CD; followed for nearly 12 years from onset.
Frequency/timing of pattern change	Observed in patients with secondary worsening after initially successful treatment; timing linked to repeated treatment sessions and loss of benefit	24/67 patients (35.8%); mean time from dystonia onset to pattern change 9.7 years; mean detection time during follow-up 52 months	37/100 patients (37%); switch occurred mainly within the first 5 years of BoNT treatment; mean time to switch 4.5 ± 2.7 years	Multiple pattern changes over time; first clear lateralized switch in 2019, about 6 years after onset and ~4 years after BoNT initiation; subsequent alternating and mixed configurations over later follow-up
Most frequent pattern/conversions	Three scenarios: unchanged direction with probable recruitment of deep muscles; same direction with changed leading muscle/pattern; opposite direction with change in leading muscle and motor program	Initial phenotype most often torticollis, followed by laterocollis; pattern change included torticollis ↔ laterocollis, torticollis → retrocollis, retrocollis → torticollis, laterocollis → retrocollis, and mixed forms	Laterocollis and rotatocollis/torticollis were the phenotypes most predisposed to change; most common conversion: laterocollis → rotatocollis/torticollis	Initial left-sided pattern with retrocollis/leftward deviation; later right-sided torticollis; then retrocollis with rightward rotation; later left-, right-, and mixed-pattern fluctuations
BoNT treatment characteristics	AboBoNT; mean dose 560 ± 325 U/session; mean 5.4 treatment sessions; mean treatment duration 1.9 years	OnaBoNT; mean 15 injections; mean dose 204 U/session overall; in pattern-change group, mean dose 248 U before vs. 247 U after change; improvement remained ~60–70%	Ongoing periodic BoNT treatment for at least 3 years; exact pooled dose summary not emphasized in the main findings; pattern changing analyzed under continuous treatment exposure	Sequential use of aboBoNT, incoBoNT, and onaBoNT; progressive dose reduction over time from higher initial doses to low-dose maintenance; sustained efficacy with longer intervals between injections

## Data Availability

Data supporting the findings of this study are available from the corresponding author upon reasonable request, subject to restrictions to protect patient privacy.

## Supplementary Materials

The following supporting information can be downloaded at https://www.mdpi.com/article/10.3390/toxins18060243/s1, Video S1. Representative clinical documentation of CD during follow-up, illustrating a left-sided dystonic pattern (January 2015), a right-sided dystonic pattern (June 2019), and a remission phase (July 2020). Table S1. Detailed longitudinal chronology of the reported case, including documented clinical follow-up visits, BoNT injection sessions, toxin formulation, total dose, target muscles, and corresponding clinical phase across follow-up.

## Abbreviations

The following abbreviations are used in this manuscript:

CD	Cervical dystonia
BoNT	Botulinum toxin
AboBoNT-A	AbobotulinumtoxinA
IncoBoNT-A	IncobotulinumtoxinA
OnaBoNT-A	OnabotulinumtoxinA
SCM	Sternocleidomastoid muscle
SPL	Splenius capitis muscle
