# Supplementary material for: Pattern Changes and Recurrent Remissions in Cervical Dystonia: Insights from a Long-Term Treated Case
Source: Toxins (Basel). 2026 May 25;18(6):243. doi: 10.3390/toxins18060243 (PMC13307584; doi:10.3390/toxins18060243)
Supplement: Supplementary file 1 [file toxins-18-00243-s001.zip › toxins-4270311-supplementary/SupTable1_CD.pdf]

Pattern Changes and Recurrent Remissions in Cervical Dystonia: Insights from a Long-Term Treated Case

Simone Aloisio, Massimiliano Passaretti, Luca Angelini, Martina De Riggi, Francesca Santachiara, Anna Sofia Grandolfo, Daniele Birreci and Matteo Bologna

Table S1. Detailed longitudinal chronology of the reported case, including documented clinical follow-up visits, BoNT injection sessions, toxin formulation, total dose, target muscles, and corresponding clinical phase across follow-up.

Timepoint	Age (years)	Main dystonic pattern	BoNT formulation	Total dose	Target muscles	Clinical phase
October 2013	30	Retrocollis with leftward deviation	None	—	—	Onset
January 2015	32	Left torticollis with mild retrocollis	AboBoNT-A	500 U	Left SPL, right SCM	BoNT initiation
April 2015	32	Persistent left-sided pattern	AboBoNT-A	700 U	Left SPL 350 U, right SCM 150 U, right levator scapulae 100 U, right SPL 100 U	Dose escalation
May 2016	33	Persistent left-sided pattern; mild dysphagia reported	IncoBoNT-A	100 U	Left SPL 50 U, right SCM 50 U	BoNT switch
September 2017	34	No clear dystonia at rest	IncoBoNT-A	100 U	Left SPL 50 U, right SCM 50 U	Near-remission
January 2018	35	No overt dystonia at rest or with activation maneuvers	None	—	—	Clinical remission
February 2018	35	No overt dystonia	None	—	—	Clinical remission
March 2018	35	Mild left laterocollis	None	—	—	Mild residual signs
April 2018	35	No overt dystonia	None	—	—	Clinical remission
June 2018	35	No overt dystonia	None	—	—	Clinical remission
September 2018	35	No clear evidence of cervical dystonia	None	—	—	Clinical remission
March 2019	36	Mild left head tilt	IncoBoNT-A	75 U	Left SCM, left SPL	Right-sided relapse
June 2019	36	Right torticollis	IncoBoNT-A	100 U	Left SCM, left SPL	Dose escalation
October 2019	37	Retrocollis with rightward rotation	OnaBoNT-A	100 U	Right SPL 70 U, left SPL 30 U	BoNT switch
January 2020	37	Persistent right torticollis	OnaBoNT-A	130 U	Right SPL 70 U, left SCM 30 U, left SPL 30 U	Dose escalation
April 2020	37	Improved right-sided pattern	OnaBoNT-A	130 U	Right SPL 70 U, left SCM 30 U, left SPL 30 U	Stable treatment
July 2020	37	Minimal rightward rotation	None	—	—	Clinical remission
October 2020	37	Minimal rightward rotation	None	—	—	Clinical remission
February 2021	38	Mild left-sided recurrence	IncoBoNT-A	70 U	Left SPL 40 U, right SPL 30 U	Left-sided relapse
May 2021	38	Left shift / left tilt	IncoBoNT-A	70 U	Left SPL 40 U, right SCM 30 U	Treatment adjustment
September 2021	38	Clinical benefit; residual left-sided pattern	IncoBoNT-A	70 U	Left SPL 40 U, right SCM 30 U	Stable treatment
December 2021	39	Clinical benefit; residual left-sided pattern	IncoBoNT-A	70 U	Left SPL 40 U, right SCM 30 U	Stable treatment

March 2022	39	No overt dystonia / minimal residual signs	None	—	—	Clinical remission
April 2022	39	No overt dystonia / minimal residual signs	None	—	—	Clinical remission
May 2022	39	Minimal rightward cervical shift	None	—	—	Mild residual signs
July 2022	39	Mild rightward shift and mild rightward rotation	None	—	—	Mild residual signs
September 2022	39	Mild right-sided pattern	None	—	—	Mild residual signs
November 2022	40	Mild rightward rotation during work activity	IncoBoNT-A	70 U	Right SPL 40 U, left SCM 30 U	Right-sided relapse
February 2023	40	Left head tilt during work activity with ipsilateral posterior tension	IncoBoNT-A	30 U	Left SPL 30 U	Dose reduction
June 2023	40	Clinical benefit; mild left-sided pattern	IncoBoNT-A	30 U	Left SPL 30 U	Stable treatment
October 2023	40	Clinical benefit; mild left-sided pattern	IncoBoNT-A	30 U	Left SPL 30 U	Stable treatment
February 2024	41	Mild left tilt with rightward rotation	OnaBoNT-A	50 U	Left SPL 30 U, left SCM 20 U	BoNT switch and dose escalation
June 2024	41	Clinical benefit; same mild mixed pattern	OnaBoNT-A	50 U	Left SPL 30 U, left SCM 20 U	Stable treatment
October 2024	41	Mild recurrence during agitation/stress; same mixed pattern	OnaBoNT-A	50 U	Left SPL 30 U, left SCM 20 U	Stable treatment
February 2025	42	Mild recurrence after ~3 months; same mixed pattern	OnaBoNT-A	50 U	Left SPL 30 U, left SCM 20 U	Stable treatment
June 2025	42	Mild recurrence after ~3 months; same mixed pattern	OnaBoNT-A	50 U	Left SPL 30 U, left SCM 20 U	Stable treatment
October 2025	42	Persistent benefit from prior injection	None	—	—	Deferred BoNT
November 2025	43	Mild recurrence with left tilt and rightward rotation	OnaBoNT-A	50 U	Left SPL 30 U, left SCM 20 U	Stable treatment

Note: In cases where muscle-specific BoNT posology is not indicated, only the total injected dose was available in the clinical documentation.
